# Metallic, carbon-based, and polymeric nanomaterials: transforming dairy farming practices for sustainability^☆^

**DOI:** 10.1016/j.fochx.2025.102640

**Published:** 2025-06-07

**Authors:** Deepti Kothari, Arun Kumar

**Affiliations:** Department of Food Science and Technology, Graphic Era (Deemed to be University), Dehradun 248002, Uttarakhand, India

**Keywords:** Carbon-based nanomaterials, dairy farming, Nanoparticles, Nanotechnology

## Abstract

Nanotechnology presents transformative solutions for the challenges facing modern dairy farming, enhancing productivity, health, and safety. This paper explores the integration of nanomaterials in dairy farming, focusing on their applications in animal health, milk production, and quality control. Metallic nanoparticles, carbon-based nanomaterials, polymeric nanoparticles, nano-emulsions, and natural nanomaterials offer innovative tools for veterinary medicine, diagnostics, and targeted drug delivery. These nanomaterials can improve nutrient absorption, hormonal regulation, and disease resistance, significantly boosting milk yield and quality. Nano-biosensors facilitate real-time monitoring of animal health and milk safety, ensuring quality standards and reducing contamination risks. However, the potential toxicity, environmental persistence, and regulatory challenges of nanomaterials warrant careful evaluation. This review paper emphasizes the need for responsible and sustainable use of nanotechnology in dairy farming, highlighting policy guidelines and recommendations for its safe and effective adoption. The findings underscore nanotechnology's pivotal role in advancing sustainable and efficient dairy farming practices.

## Highlights

• Nanotechnology supports livestock drug delivery and disease diagnostics.
• Nano-interventions improve milk yield, quality, and safety.
• Graphene-based sensors aid in rapid pathogen detection.
• Nano-ZnO improves udder health and controls mastitis.
• Nanotech offers innovative tools for dairy animal health management.

## 1 Introduction

Dairy farming has a significant impact on the rural economy. Milk has become a significant way to earn money in rural India. Milk and various dairy products play a key role in our everyday diets. India leads the globe in both the production and consumption of milk. It also boasts the largest dairy herd in the world, which includes water buffalo and native as well as crossbred cattle (Vekariya et al., 2021). Dairy farming encounters significant challenges, especially related to environmental sustainability, animal welfare, changes in farm structures, and fluctuations in prices for inputs and outputs. These issues created a great opportunity for future research in the economics of dairy production (Bravo-Ureta et al., 2021). The main stakeholders in the dairy industry include milk producers, milk processors, marketers, retailers, and consumers. Milk producers, the value chain, and consumers all play a crucial role in the growth of the dairy industry (Sarkar & Dutta, 2020).

Nanotechnology in the food industry, particularly in dairy, was always advancing. There was a lot of research focused on how to process raw materials efficiently, develop functional products, and improve the preservation, packaging, and storage of finished goods (Smykov, 2020). Around the world, agricultural systems face increasing challenges from unforeseeable endanger. To protect maintainable farming and food manufacturing, using advanced nanotechnology in plants, known as phytonanotechnology. It can boost farming by reducing losses and making better use of resources (Jiang et al., 2021). This offers a key solution for maintaining the sustainable growth of agricultural systems and related industries. Baig et al. (2021) described nanomaterials (NMs) as having internal surface structures or external sizes that measure between 1 and 100 nm in one or two dimensions. NMs play a key role in the precise transport of proteins, nucleotides, and other active plant molecules. Several kinds of nanoparticles (NPs) were commonly used in phytonanotechnology. These include quantum dots (QDs), mesoporous silica NPs (MSNs), metallic NPs, carbon nanotubes (CNTs), magnetic NPs (MNPs), and metal oxide NPs (Jiang et al., 2021). On the other hand, Vejan et al. (2021) demonstrated that different kinds of nano-fertilizers, such as controlled-release and slow-release types, were made using innovative methods. These include materials that were polymer-coated or encapsulated, as well as those based on zeolite and biochar. It is growing because of their coating layers that come from natural sources, and well-organized release helps deliver nutrients to the intended plants, which in turn promotes quicker germination, faster growth, and a high level of nutrient absorption. Right now, the farming community is dealing with many problems like low crop yield, damage to plants from pests, reduced soil quality, etc. To solve these problems, nanoagroparticles can effectively work as fungicides, insecticides, herbicides, and pesticides (Baker et al., 2017). Kim et al. (2018) also stated that nanoformulations that include standard antibiotics, especially tetracycline, demonstrated a greater ability to fight against antibiotic-resistant plant pathogens. On the other hand, Nano-biofertilizers (NBFs) were reported as the most effective alternative to conventional fertilizers, a mixture of biofertilizers, organic materials, and NMs. It releases nutrients in a slow and controlled way, which allows plants to get the nutrients they need gradually throughout their growing season (Ali et al., 2021). The findings revealed that both silver (Ag) and copper (Cu) monometallic graphene composites can reduce bacterial growth. However, the Ag-based graphene composite had a stronger antibacterial effect than the Cu-based one (Kim et al., 2018).

Since, agriculture farming supports dairy farming by providing essential feed and fodder for livestock, it becomes essential to manage agricultural practices for improved dairy farming. Breed management was a costly and time-demanding issue for farmers. Mekonnen (2021) deciphered that inserting a nanotube under the skin to measure changes in the amount of estradiol in the blood in real time. Nanotubes were used to monitor oestrus in animals because they can attach to and identify the estradiol antibody during oestrus using near-infrared fluorescence. NPs might play an important role in managing insect pests and harmful germs.

Sustainability presents both challenges and opportunities, so food and dairy companies need to rethink their strategies for achieving sustainable success. These practices include conserving energy, reducing emissions, redesigning systems, managing waste, and recycling. By doing so, they can improve both economic and social performance (Shoaib et al., 2022). Sustainable agriculture means managing and using farming systems in a way that keeps biological diversity, the ability to regenerate, health, and productivity both now and in the future (Arvidsson Segerkvist et al., 2020). Kozina and Semkiv (2020) stated that the dairy industry was one of the earliest livestock sectors to adopt a smart production management system. It included systems for identifying animals. Whereas, Brito et al. (2021) reported the effects of nitrogen emissions from dairy cows and focus on emissions related to environmental science, farming efficiency, and following the law. There are limited studies that describe the applications and benefits of nanotechnology in dairy farming. Therefore, this review explores the benefits and applications of sustainable dairy farming practices using different nanomaterials.

### 1.1 Types of nanomaterial used in dairy farming

#### 1.1.1 Metallic nanoparticles

**Fig. 1:**
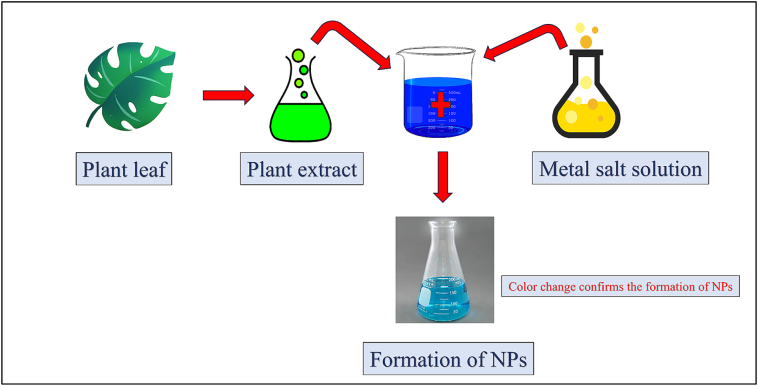
Process for formulation of nanoparticles.

**Fig. 2:**
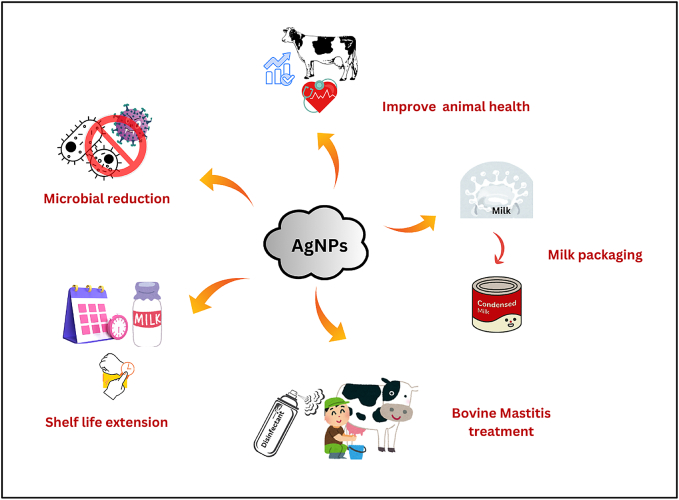
Application of silver nanoparticles in dairy.

Mastitis is a serious problem for the dairy industry worldwide. *S. aureus* was the most common pathogen that causes mastitis. Subclinical mastitis was said to significantly affect the finances of farms and the well-being of animals. At the same time, it reduced both the amount and quality of milk, making it unsuitable for processing (Lodhi et al., 2021). The overview of and benefits and applications of metallic nanomaterials is summarized in Table 1. Green synthesis of nanoparticles using plant extracts is an eco-friendly and sustainable approach that utilizes phytochemicals as natural reducing and stabilizing agents (Fig. 1). Earlier research by Kalińska et al. (2023) suggested that CuNPs, AgNPs, and AgCuNPs were innocuous for human and bovine mammary gland cells and reduced their potentiality of *E. coli* and *Staphylococcus aureus*. The applications of AgNPs in dairy industries are shown in Fig. 2. Additionally, the blend of AgNPs and CuNPs can reduce the survival rate of pathogens and has strong germ-fighting abilities, which can nearly eliminate bacterial biofilm. Most of the Zn in animal diets comes in two types: organic Zn, like Zn -amino acids, and inorganic Zn, such as zinc oxide (ZnO) and zinc sulfate (ZnSO_4_). Adding ZnNPs at doses of 100 and 200 mg/kg has boosted the levels of volatile fatty acids, microbial crude protein, and the breakdown of organic matter after 6 and 12 h of incubation period under *in vitro* condition that stimulates rumen fermentation (Abdelnour et al., 2021). Whereas AgNPs can be used to prevent and treat the infectious bursa disease virus. It boosted the adaptive immune system and can help in speedy healing of wounds, surgical cuts, and burns. It also has the ability to enhance the effectiveness of existing medications, even when used at lower doses (Adegbeye et al., 2021). Akgönüllü et al. (2021) developed a plasmonic sensor that used molecular imprinting to directly find a small amount of AFM1 in a raw milk sample. They developed a polymer nanofilm that includes AuNPs on a gold plasmonic sensor chip that was covered with allyl mercaptan and allowed for the diagnosis of AFM1 in milk. The earlier studies by Amor et al. (2024) have also shown that adding chitosan to the surfaces of metallic NPs can bring several benefits, including better stability, serving as a drug carrier, controlling drug release, improving tissue penetration, boosting cell interaction, and enhancing anti-microbial activity. Couto and Almeida (2022) studied the effectiveness of low-density polyethylene-Ag nanocomposites in protecting food from biofilm-forming *E. coli*, and helped store items and serve as general-purpose containers.

**Table 1:** Overview of applications of different nanomaterials in dairy industry.

**Nanomaterial type**	**Benefits**	**Major findings**	**References**
Silver	It boosted gut bacteria, raised the level of antibodies, and lowered the death rate.	Showed stronger antibacterial effect.	Kim et al. (2018)
	It speeds up the healing of wounds, surgical cuts, and burns.	Boosted immune system, can be used to prevent and treat the infectious bursa disease virus.	Adegbeye et al. (2021)

Copper	It has antibacterial and antifungal effects.	CuNPs may influence or decrease pathogens' viability and may be used in mastitis treatment.	Kalińska et al. (2023)
	It improved catalase enzyme activity, immunoglobulin M (IgM) and (IgA).	It reduced or retard the bacterial growth.	Kim et al. (2018)

Zinc	It improved animal growth, better feed efficiency, and stronger immune responses.	It helped enhance cow's udder health and diminish the milk somatic cell count (SCC).	El-Sayed and Kamel (2021)
	It showed antifungal properties,	It was helpful in the elimination of ringworm infections and could serve as an active ingredient in skin treatments.	Djearamane et al. (2022)

Gold	It linked with aptamers (nanoaptasensors) to detect antibiotics used in cattle through colorimetric detection in raw milk.	It used to detect four antibiotics: kanamycin, oxytetracycline, sulfadimethoxine, and ampicillin.	Díaz-García et al. (2022)
	Gold nanoparticles combined with carbon nanotubes	It can detect thiamphenicol residues in milk samples.	Muhammad et al. (2018)

Graphene Oxide	N/A	Detection of aflatoxin compounds, aflatoxin M1 and its derivatives, aflatoxin B1	Tanveer et al. (2023)

N/A: Not available.

Calcium oxide can also be used in dairy farming because it is not harmful and remains stable in harsh environments. It can produce reactive oxygen species (ROS), which lead to cell death (Srilekha et al., 2022). Nano-Cu and nano-Zn can boost the activity of the superoxide dismutase enzymes, which play a key role in protecting the body from oxidative stress as an important antioxidant defense. Nano-Se is also known to boost the activity of this enzyme, enhance the efficiency of the antioxidant system, and prevent oxidative stress (Michalak et al., 2022). The study also highlighted that Zn-NPs can influence various aspects of animal health and performance, including growth, milk production, rumen fermentation, immune function, reproduction, antibacterial effects, and potential toxicity.

#### 1.1.2 Carbon-based nanomaterials

**Fig. 3:**
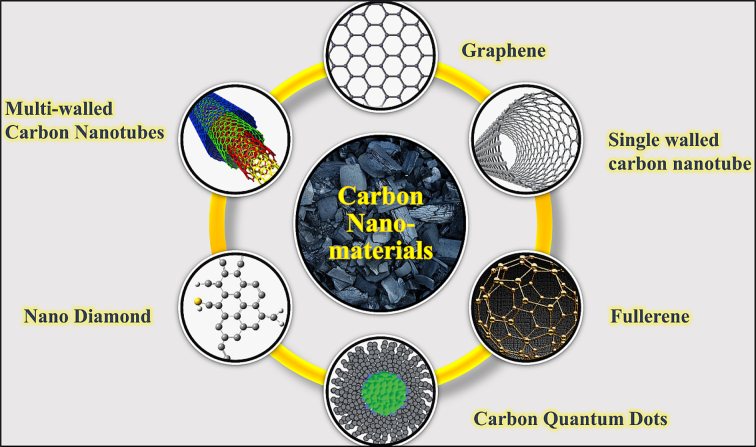
Different carbon nanomaterials used in dairy.

**Fig. 4:**
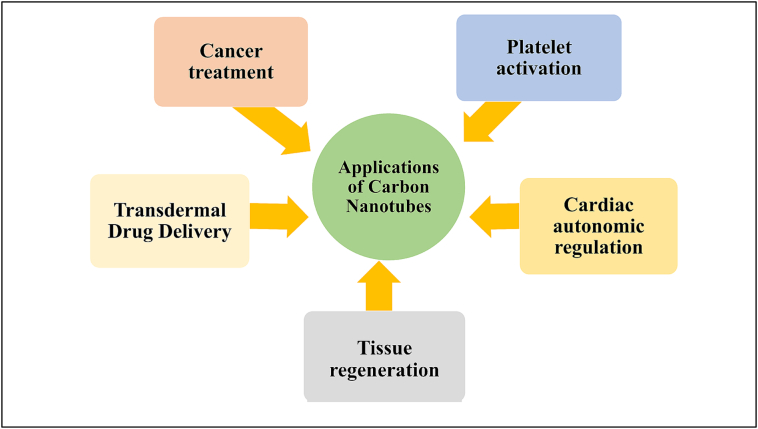
Application of carbon nanotubes.

CNPs are categorized into different types based on their size and shape (Fig. 3). It included zero-dimensional NPs like fullerenes and CQDs, one-dimensional NPs such as carbon nanofibers and CNTs, two-dimensional NPs like graphene, and three-dimensional NPs, which include carbon sponges (Dutta et al., 2022). Graphene oxide (GO) has been employed in the development of biosensor prototypes for aflatoxin detection, as demonstrated by Gao et al. (2021), who utilized GO due to its favourable physicochemical properties and functional surface chemistry. The study concluded that graphene-based sensors can be used for the identification of mycotoxins in milk. The sensors were made by combining reduced graphene oxide (rGO) with AuNPs. Biosensors made from graphene were used in farming to detect microorganisms in food, monitor crop health, and check for signs of illness in animals. It offers several benefits, like better consistency between different sensors and longer shelf life, decreased biofouling, preservation of sensor functions, and a lower chance of the enzyme unfolding (Chick et al., 2024). Also, CNTs can identify specific DNA sequences, cancer markers, and viruses as shown in Fig. 4 (Hassan et al., 2020).

#### 1.1.3 Polymeric nanoparticles

Polymeric nanoparticles (PNPs) were made from bio-reasonable and environment friendly materials like PLGA (Poly lactic-*co*-glycolic acid) (Gonçalves et al., 2021). PNPs can be divided into two primary categories: nano capsules and nanospheres. Nano capsules serve as storage units for holding drugs within a liquid, either watery or oily, inside the core of capsules, which were surrounded by a solid shell made of polymer. Nanospheres are solid polymer structures where molecules are either captured in the center of the sphere or attached to the surface of the NPs (Al-Nemrawi et al., 2022). In a study, Yadav et al. (2022), evaluated chitosan (natural polymer) for its potential in the formulation of nanomedicines aimed at the prevention and management of mastitis. Due to its excellent drug delivery properties and water solubility, chitosan was recognized as an effective carrier. Its mucoadhesive nature facilitates enhanced drug penetration by adhering to mucosal surfaces, and it can improve the epithelial permeation of macromolecules by transiently opening tight junctions.

#### 1.1.4 Nanoemulsions

Nanoemulsions are thermodynamically unstable mixtures that combine at least two non-blendable liquids, usually oil and water, as well as stabilizers like emulsifiers, texture modifiers, ripening inhibitors, and weighting agents, with a droplet size below 100 nm (Ozogul et al., 2022). These were developed using methods that involve pressure or energy, such as high-pressure homogenizers or microfluidizers (Panghal et al., 2019). The use of nanoemulsions was demonstrated in the study by Salama et al. (2022), in which essential oil (EO) nanoemulsions were prepared and subjected to thermal stability testing to evaluate potential oil separation during the storage of EO-enriched cultured milk intended for the production of flavored stirred yogurt (SY). The findings revealed that all nanoemulsions remained stable after the treatment and showed no signs of oil separation. Qazi et al. (2022) stated that all the recombined milk systems created curd under gastric conditions, which slowed down the release of protein and oil droplets containing curcumin. The heat caused the casein and whey proteins to form complexes, which led to a loose, broken curd when the reconstituted powder was treated with high heat and allowed more protein and oil droplets to pass into the intestine. Heydari Gharehcheshmeh et al. (2021) indicated that adding a nanoemulsion of sweet almond and sesame oil with an emulsifier lowered the pH and syneresis and raised the acidity, malondialdehyde levels, and antioxidant activity. While adding sesame and sweet almond oils, the yogurt viscosity retarded but the elastic modulus and viscous modulus values went up. Nanoemulsions were used in animal nutrition to boost the bioavailability, stability, and effectiveness of different feed additives and also increased the absorption of fat-soluble compounds like vitamins, fatty acids, and essential oils (Salama et al., 2022).

## 2 Application of nanomaterials in animal health

### 2.1 Nanomaterials in veterinary medicines

NPs that help in discovering and treating diseases, delivering medicines, and supporting animal breeding and reproduction, include QDs, nanopores, magnetic nanoparticles, nanoshells, polymeric NPs, fullerenes, liposomes, and dendrimers (Woldeamanuel et al., 2021). Albumin is an animal protein that has been recognized for its various medical uses. It helps in medical treatments, and was also used in different mixtures to deliver medicines and diagnostic tools, and Abraxane, a type of medicine made from albumin nanoparticles that help in treating cancer in the future (Kianfar, 2021). The use of Polybutylcyanoacrylate (PBCA) as a pulmonary administrator was studied by Luo et al. (2021) and revealed that it was pH delicated and can be prompted by enzymes. PBCA-encapsulated DOX immensely expanded the drug consumption of lung cancer cells, boosted the anti-cancer activity of drugs, and raised the likelihood of survival of animals. Anwar et al. (2021) developed DNA vaccines, polynucleotide vaccines, plasmid vaccines, and gene-based vaccines that send specific genes to host cells where they make protein antigens near special cells that showed these antigens to the immune system, kicking off the immune response. Liu et al. (2023) also developed a new type of microsphere that has a special hydrogel shell made of alginate and calcium, which targets the colon and reduced acid. The core of the microsphere was made from thiolated-hyaluronic acid, giving it a strong ability to stick to surfaces in the body.

### 2.2 Antimicrobial nanoparticles for disease prevention

**Fig. 5:**
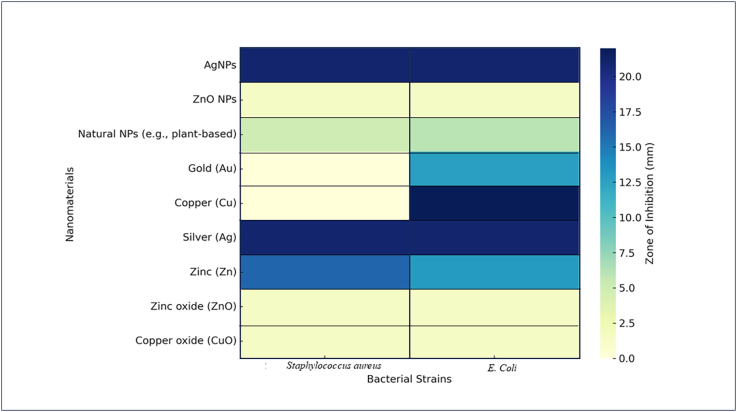
Heatmap illustrating the antibacterial activity of different nanomaterials against *Staphylococcus aureus* and *E. coli*, based on their zones of inhibition.

El-Sayed and Kamel (2021) reported that Nano-ZnO can help dairy cows with subclinical mastitis by enhancing their udder health and diminishing the milk somatic cell count (SCC). Their strong ability to get rid of pathogens that cause mastitis, along with being safe for mammal cells and affordable, makes them suitable for treating mastitis. The antimicrobial properties of chitosan were greatly influenced by its molecular weight, the kind of organisms, the pH level, the polymer's degree of polymerization, and the presence of lipids and proteins on the surface of the microbes. It binds to the bacterial surface, which caused to agglutination, making their walls more permeable, and eventually leads to the spilling of their internal substances (Kalashgarani & Babapoor, 2022). Furthermore, AgNPs have the same ability to fight against bacteria. AgNPs can stick to the cell wall and break through it. This action caused changes in the cell membrane structure, affecting its permeability and ultimately resulting in cell death (De Silva et al., 2021). Also, the proposed way that AgNPs work involves causing cell death by producing ROS in certain bacteria, such as *E. coli, Klebsiella pneumoniae,* and *Pseudomonas aeruginosa* (El-Gohary et al., 2020). Yusof et al. (2022) reported that ZnO NPs were very effective at fighting bacteria. It can help improve their growth and performance, while also acting as an alternative antibacterial method to manage diseases. Researchers also looked into the use of bacteriocins as substitutes for antibiotics. Bacteriocins are peptides produced by bacteria that can fight against harmful microbes. Recently, treatments that include bacteriocins produced by *Streptococcus* have been developed to treat mastitis (El-Sayed & Kamel, 2021). The heatmap illustrating the antibacterial activity of different nanomaterials as published in few literarures is shown in Fig. 5 (Dadi, Azouani, Traore, Mielcarek and Kanaev, 2019, Hamed and Emara, 2023, Tavares et al., 2020).

### 2.3 Biosensors for real time health monitoring

Alipio and Villena (2023) highlighted the application of biosensor devices and smart wearable technology in their study and stated that biosensors measure temperature. It merges a biological component, like antibodies or pathogens, with electronic components to create a signal that can be detected and measured. Whereas, few wearable wireless biosensors have been developed and studied by Lee and Seo (2021), and stated that the rumen bolus system was inserted orally and then positioned in the reticulum to keep track of some rumen factors, like temperature and pH.

Three diverse types of biosensors can be used to detect the jaw movement that designates cattle grazing behavior. It includes pressure sensors, electromyography sensors, mechanical sensors, and acoustic sensors. It analyzes the posture of the cow, the cow's motion, position, and jaw movement (Džermeikaitė et al., 2023). Another example of biosensors was studied by Khan (2022) and stated that an electrochemical biosensor (ECB) was a sensing device that relies on the chemical reaction between immobilized biomolecules and target analytes that turn biochemical information into signals that can be analyzed. Researchers tested a minimally invasive technique that uses implantable biosensors with radio-frequency identification (RFID) technology. This method was examined for its ability to check the subdermal temperature at the ear base of a dairy cow (Chung et al., 2020). Recently, the use of Internet of Things (IoT) was studied by Karthick et al. (2020) and revealed that Internet of Things in Animal Healthcare (IoTAH) uses biosensors and software to track and keep animal health records. These technologies accurately assess health conditions and predict illness. Poudineh et al. (2021) combined aptamers and antibodies in a bead-based fluorescence sandwich immunoassay that was set up in a specially designed microfluidic chip and employed the assay to continuously measure glucose and insulin levels in the blood of living organisms.

### 2.4 Detection of pathogens in livestock

Biosensor test uses a receptor that identifies the infectious agents and a transducer that senses the signal indicating the presence of the disease. Gamma interferon assays helped identify tuberculosis in primates, cattle, and cervids (Suminda et al., 2022). To detect pathogens like *Salmonella*, Kumar et al. (2020) used single-walled surface carbon nanotubes (SWCNTs) in a DNA sensor. This was done by covalently attaching N-ethyl-N′-(3-dimethylaminopropyl) carbodiimide hydrochloride to the nanotubes. Metagenomic NGS has been used to find infrequent and new viruses or to explain the variety of viruses in samples from humans, animals, and the environment (Suminda et al., 2022).

### 2.5 Enhanced immunity and reduced disease outbreak

NPs had demonstrated strong possibilities in various areas, such as medicine, creating vaccines, and can be used to improve the effectiveness of vaccines for viral diseases in animals (Kumar et al., 2024). Many viruses have been utilized to develop virus-vectored vaccines that offer strong protection against antigens. The Newcastle disease virus (NDV) can be used to create a vaccine, and recombinant NDV (rNDV) vaccine strains were very safe because they only target specific hosts and replicate in the cytoplasm (Allam et al., 2023). A study by Yao et al. (2023) demonstrated the use of live reduced vaccines, S19 and RB51 vaccines, for protecting against bovine brucellosis. The attenuated antigens in live reduced vaccines can still replicate, allowing for long-lasting immunity. Another use of DNA plasmid vaccines was studied by Vuppu et al. (2024) and reported that it works well against the avian influenza virus, lymphocytic choriomeningitis virus, etc. in animals. Whereas, polymer-based nanovaccines provide a steady release of antigens, keep these antigens stable, and boost immune responses from both cells and antibodies. To enhance cow's vaccinations, researchers used chitosan NPs (silica NPs, and polylactic acid-glycolic acid (PLGA) NPs as adjuvants or carriers for the vaccines. The chitosan-coated PLGA DNA NP vaccine (chi-PLGA-DNA), boosted mucosal, systemic, and cell immunity in cattle. It decreased the severity of the disease, slows down the virus, leaves the body, and delays the start of symptoms (Rehman et al., 2024).

## 3 Enhancing milk production and nanotechnology

### 3.1 Role of nanomaterials in optimizing animal nutrition

Nano Zn helped animals absorb Zn better, which can lead to improved growth, better feed efficiency, stronger immune responses, enhanced antioxidant levels, and higher quality of meat (Almeida et al., 2024). Dumlu (2024) reported that nanoparticle minerals used as feed additives can boost bioavailability by moving through the intestinal wall to body cells more quickly, and enhance animal productivity, boost digestion and absorption of feed. Among the various types of NPs, Bhagat and Singh (2022) reported CuO NPs, ZnO NPs, Selenium (Se) NPs, and cerium oxide (CeO_2_) NPs for their ability to fight microbes and provide antioxidant benefits. Whereas CuO NPs had boosted immune defense and improved antioxidant capabilities, increased catalase enzyme activity, and immunoglobulin M (IgM) and IgA. Adding nanosilver to feed helped boost their gut bacteria, and strengthens their immune and physical responses. It raised the levels of antibodies against the Newcastle disease virus and lowered the death rate and injury points (Almeida et al., 2024). Similar studies by Abdelnour et al. (2021) also illustrated that Se had great bioavailability because they had high catalytic efficiency, low toxicity, and a strong ability to absorb solids. Whereas, AgNPs helped promote growth in animal nutrition and altered the gut bacteria. It helped animals grow better, boosted their immune system, digestion power and retarded their death rates (Al-Sultan et al., 2022).

### 3.2 Nanofortified feed additives

Ghosh et al. (2022) stated that fortification of foods by adding health-boosting and nutrition-enhancing ingredients has been a popular way to improved their benefits for a long time. The work done by Raya et al. (2016) reported that biscuits containing nano ZnO were used as a food supplement to help manage and control Zn deficiency diseases. Results showed that they experienced a quick boost in their growth rate, appetite, and hair growth. In a study by Hashem and Gonzalez-Bulnes (2021), also reported that the process of nano-emulsifying soybean and fish oil, which focused on delivering long-chain polyunsaturated fatty acid, greatly boosted the levels of these fatty acid like oleic, linoleic, and linolenic acids in the rumen fluid and also used solid lipid nanoparticles made of arachidonic or stearic acids to shield lysine from being broken down by the enzymes in ruminal microbiota.

### 3.3 Nanoparticles for reproductive health and hormonal regulation

The reproduction rate of farm animals greatly affects the profits and productivity of livestock farming. Using various methods like diet changes, hormone treatments, and biological approaches was key to effective reproductive management (Gulzar et al., 2024). Hashem and Gonzalez-Bulnes (2021) proved the effectiveness of chitosan-TPP nanoparticles for delivering nasal human chorionic gonadotropins (hCG) to help trigger ovulation in cows. Gulzar et al. (2024) recommended antibiotic-based treatments for many diseases caused by microbes and protozoa, including those related to reproduction. Whereas Omar et al. (2025) focused on the efficacy of gonadotropin-releasing hormone-loaded chitosan-TPP nanoparticles (GnRH-CNPs) and prostaglandin F2α-loaded chitosan-TPP nanoparticles (PGF2α -CNPs) in improving reproductive success in dairy cows that were under heat stress. A recent study by Hashem and Gonzalez-Bulnes (2021) examined the efficacy of novel 3-molecule boar pheromone spray to enhance breeding success. The rise of aerosol nano-drug delivery technology paved the way for creating many pheromone-based methods to manage reproductive events like male effects, estrous detection, pregnancy diagnosis etc. In another study, they used a nano-based drug delivery system to provide GnRH and PGF2 α in animals. The findings highlighted that GnRH and PGF2α can enhance the ovary's response, blood flow patterns and hormone levels during the Ovsynch protocol after every injection (Hashem et al., 2022).

### 3.4 Reduction of mastitis and other milk production challenges

A study by Hashem and Gonzalez-Bulnes (2021) demonstrated that using amoxicillin NPs to treat bovine mastitis could extend the effects of the antibiotic after treatment, allowing for longer time periods between doses. According to Bhakat et al. (2020), using antiseptics on teats after milking was the best way to prevent intramammary infection (IMI) in lactating cows, and giving tri‑sodium citrate to lactating cows was a potent, simple, and affordable way to reduce sub-clinical mastitis in cows. In another study, scientists developed automatic perception methods for bovine mastitis using biosensors. These methods used suitable sensing technology, such as in-line monitoring of somatic cell count (ISCC) and checked the electrical conductivity (EC) of milk to find mastitis (Neculai-Valeanu & Ariton, 2022). The primary way to treat mastitis was with antibiotics like penicillin, ampicillin, tetracycline, and gentamicin that can be administered through an infusion directly into the mammary gland or given as injections into the muscle or veins (Cheng & Han, 2020). Hassan et al. (2020) developed a polydimethylsiloxane film coated with reduced graphene oxide (GO) for a hearing transmitter. It was used to generate high-pressure and high-frequency ultrasound, which helped in diagnosing and treating diseases in both humans and animals.

## 4 Nanotechnology for milk quality and safety

### 4.1 Adulteration of milk

Milk and milk-based products were an important part of diets, so their contamination or adulteration, like biological ones, including microbes or their toxins, chemicals like urea and melamine, was a serious concern (Singh et al., 2021). Melamine contamination in food and medicine wai0 major issue. Singh (2023) used surface-enhanced Raman spectroscopy for the fast detection of melamine in milk and milk products. According to a study by Kalpana et al. (2020), they used the Optoelectronic Testing method for detecting melamine adulteration in milk with para-Nitroaniline modified AgNPs,and could also detect by developing AgNPs using tannic acid directly in the sample. Varun et al. (2017) reported that sodium D-gluconate with AuNPs and AgNPs can be used for detecting melamine. In perspective of this, Singh et al. (2020) also highlighted that AuNPs can be a quick test for the detection of melamine. It relies on the way melamine caused AuNPsto clump together, which changed their color from red to blue. For the detection of urea in milk, enzymatic and aptamer-based biosensors were used. Usually, the bioreceptor used for detecting urea was urease, and it helped break down urea into carbon dioxide and ammonia (Shalileh et al., 2023). In another study, a colorimetric sensor made from a zein/ manganiotese dioxide nanosheet(MnO_2_ NS) composite was created. This sensor worked with a smartphone and was designed for quick and accurate testing of hydrogen peroxide (H_2_O_2_) and lactic acid (Pereira et al., 2024).

Flurophores like organic dyes, carbon and graphene quantum dots (CDs and GrQDs) and semiconductor quantum dots were utilized in fluorescent sensors and they serve as fluorescent probes to help find adulterants in milk (Singh, 2023). Angelopoulou et al. (2021) used a silicon-based photonic immunosensors to detect mozzarella and feta cheese in milk samples. The photonic immunosensor used Mach-Zehnder interferometers that were built together with their light sources on a silicon chip. Another study by Aftab et al. (2023) used AuNPs with graphene hydrogel nanocomposites (AuNPs-GHs) to detect IAA (indole-3-acetic acid) and salicyclic acid through chrono-amperometric measurements.

### 4.2 Nano-biosensors for quality assessment

Aflatoxins are a group of harmful and cancer-causing substances that can be present in food and used AuNPs that were coated with anti-aflatoxin antibodies to detect aflatoxin B1. The method using AuNPs in an immunochromatographic strip, has been used to detect aflatoxin M1 in milk (Kumar et al., 2017).

### 4.3 Reduction of antibiotic residue in milk using nanocarriers

Electrochemical sensors focuses on electrode surfaces made from Au and carbon, including graphite, carbon black (CB), multi-walled carbon nanotubes (MWCNT) to detect antibiotic residues in milk (De Faria et al., 2021). Gutiérrez et al. (2020) had previously reported a new method using AuNPs for detecting antibiotics in milk including Sulfadimethoxine, Kanamycin, and adenosine made with AuNPs and aptamers. In parallel, Jing et al. (2021) developed a highly sensitive SERS platform using Ag/TNA along with SPME to detect the antibiotic breakdown product in milk. Vuran et al. (2021) used a method that combines magnetic solid phaseextraction (MSPE) with high-performance liquid chromatography-diode array detection (HPLC-DAD) to measure the amount of chloramphenicol (CP) and tetracycline (TET) antibiotic residues in milk sample. Whereas, an integrated amperometric immunosensor was developed by Singh et al. (2023) that used a screen-printed carbon electrode to detect sulfonamides in milk and an affinity magnetosensor, which used screen-printed carbon electrodes and a recombinant penicillin-binding protein has been developed to detect β-lactam residue in milk.

On the other hand, biosensors based on electrochemical aptamers that combined magnetic nanoparticles (MNP) and AuNPs to identify tetracycline residues (Singh et al., 2023).

## 5 Challenges and risks associated with nanomaterials

### 5.1 Potential toxicity of nanomaterials to animals and human

Nanotoxicology focused on how nanomaterials can negatively impact living things and the environment. The biological effect of nanomaterials usually rely on their size and shape (Van der Merwe & Pickrell, 2018). The common use of NPs raises worries about their toxicity to the male and female reproductive systems and fetal health. This concern was heightened because NPs are very small and can easily enter the body, and can also pass through the placenta, which can harm the fetus and lead to toxicity (Brohi et al., 2017).

In another study, Gulati et al. (2022) concluded that CNTs can be found in the air and sometimes group together in fuel gas streams. This can pollute the atmosphere and may even enter the lungs Theoxidative stress, which is triggered by free radicals play a major role in many illnesses. NPs also greatly increased cell death by using oxidative stress mechanisms that harm DNA in mammal cells (Brohi et al., 2017). On the other hand, Hassan et al. (2020) investigated that after injecting CNPs suspension injection into animals, they gain body weight, their blood and chemical levels remain the same, and the oxidative stress caused by CNMs had a major toxic impact on animals and led to health issues. However, GO caused serious lung damage, which included higher levels of proinflammatory cytokines and apoptotic cells in the bronchoalveolar lavage fluid (BALF), as well as a breakdown of the alveolar-capillary barrier (Ema et al., 2017). Further researchers examined the potential harmful effects of pure MWCNTs and reported that they caused more harm, like destroying cells and triggering programmed cell death (Chen et al., 2018). Some NPs had adverse effect on animals like inflammation, ulceration, and decreased in growth rate, decline in viability, and triggering neurobehavioral alteration in animal models (Malhotra et al., 2020) whereas, GO NPs may adsorb antibiotics such as levofloxacin, manipulating their mobility, transport and effect, increasing their risk of toxicity.

### 5.2 Environmental persistence and ecological impact of nanomaterials

Gulati et al. (2022) reported that NMs can also harm the air system. The NMs can also form dust clouds after they were released into the environment. The use of NMs can also lead to harmful effects on the environment and ecosystems. Many NMs were inevitably released into lakes, rivers, and oceans, which can affect drinking water and both aquatic life and humans (Saleem & Zaidi, 2020). The study demonstrated that CNTs do not dissolve in water, and settle in sediments. This was harmful to bottom-dwelling animals in water and also affected the movement of other pollutants that were present. Silicon dioxide NMs can enter the atmosphere during volcanic eruption. When these particles were in the air, they can irritate 9 nm the eyes (Malakar et al., 2021). Saleem et al. (2021) found enough proof that CNTs can harm human health. They noted issues like breathing problems and lung scarring related to longer, straighter CNTs structures.

Natural nanomaterials (NNMs) from volcanic ash can easily travel to various surfaces like lakes, rivers, seas, and oceans through wind and rain, which may raise the risk of toxicity (Malakar et al., 2021). However, the undesirable effects of NMs on human health, ecology, and environment have been discussed by Pérez-Hernández et al. (2021) and stated that TiO_2_-NPs produced ROS and caused changes in tissues, cancer development, damage to genetic materials, and problems with the immune systems. The harmful effects of NNMs mainly come from their breakdown and the release of metal ions. This happened often in low-oxygen environments and relies on how quickly they dissolved in conditions that were relevant to the environment (Malakar et al., 2021).

### 5.3 Regulatory challenges in nanotechnology adoption

Regulatory policies that ensure the safety of NPs were essential for keeping people healthy. These policies reduced environmental risks by evaluating and controlling how NMs affect ecosystems, biodiversity, and the environment (Isibor, 2024). In parallel study by Yadav et al. (2023) revealed that the use of precision farming methods that involve nanotechnology, farmers can cut down on the use of agrochemicals. This approach helped them keep their crop yield high, and protects the health of soil and water. As earlier mentioned, dairy farming is indirectly supported by agriculture farming, the regulatory challenges prevails. Because of the possible dangers of NMs, many developed countries have actively participated in international groups like the Organization for Economic Co-operation and Development. These global organizations have been developing guidelines and standards for testing NMs (Park & Yeo, 2016).

## 6 Conclusion

The application of metallic, carbon-based, and polymeric nanomaterials in dairy farming marks a significant step toward achieving sustainability and efficiency in the sector. These advanced materials offer innovative solutions to long-standing challenges, enhancing animal health, productivity, environmental management, and product safety. Metallic nanomaterials such as silver, copper, and zinc oxide possess antimicrobial properties, contributing to improved hygiene, reduced disease incidence, and minimized antibiotic use. These benefits directly impact animal welfare and milk quality, supporting safer dairy practices. Carbon-based nanomaterials, including graphene and carbon nanotubes, bring exceptional sensing capabilities. Their integration into biosensors allows real-time monitoring of animal health parameters, early disease detection, and precise nutrient delivery. Such smart technologies contribute to precision dairy farming, where resources are optimized, and productivity is maximized. Additionally, carbon nanomaterials play a role in environmental management, such as adsorbing pollutants and improving waste treatment. Polymeric nanomaterials further enhance sustainability through controlled drug delivery, biodegradable packaging, and nutrient encapsulation. These features help reduce environmental load, ensure effective treatment of livestock, and extend product shelf life. Their role in developing smart packaging also supports food safety and traceability in dairy products. Despite these advancements, the safe and responsible use of nanomaterials must be emphasized. Concerns regarding toxicity, accumulation in food chains, and regulatory gaps require focused research and clear guidelines.

In summary, metallic, carbon-based, and polymeric nanomaterials offer transformative potential for sustainable dairy farming. Their responsible integration can lead to enhanced productivity, reduced environmental footprint, and improved animal and consumer health, aligning with modern agricultural and ecological goals.

## CRediT authorship contribution statement

**Deepti Kothari:** Writing – original draft, Data curation. **Arun Kumar:** Writing – review & editing, Supervision, Formal analysis.

## Declaration of competing interest

The authors declare that they have no known competing financial interests or personal relationships that could have appeared to influence the work reported in this paper.

## Data Availability

Data will be made available on request.

## References

[bb0005] Abdelnour S.A., Alagawany M., Hashem N.M., Farag M.R., Alghamdi E.S., Hassan F.U., Elwan H.A. (2021). Nanominerals: Fabrication methods, benefits and hazards, and their applications in ruminants with special reference to selenium and zinc nanoparticles. Animals.

[bb0010] Adegbeye M.J., Elghandour M.M., Reddy P.R.K., Alqaisi O., Oloketuyi S., Salem A.Z., Asaniyan E.K. (2021). Silver nanomaterials for agri-food applications.

[bb0015] Aftab R., Ahsan S., Liaqat A., Safdar M., Chughtai M.F.J., Nadeem M., Khaliq A. (2023). Green-synthesized selenium nanoparticles using garlic extract and their application for rapid detection of salicylic acid in milk. Food Science and Technology.

[bb0020] Akgönüllü S., Yavuz H., Denizli A. (2021). Development of gold nanoparticles decorated molecularly imprinted–based plasmonic sensor for the detection of aflatoxin M1 in milk samples. Chemosensors.

[bb0025] Ali S.S., Al-Tohamy R., Koutra E., Moawad M.S., Kornaros M., Mustafa A.M., Jiao H. (2021). Nanobiotechnological advancements in agriculture and food industry: Applications, nanotoxicity, and future perspectives. Science of the Total Environment.

[bb0030] Alipio M., Villena M.L. (2023). Intelligent wearable devices and biosensors for monitoring cattle health conditions: A review and classification. Smart Health.

[bb0035] Allam A.M., Elbayoumy M.K., Ghazy A.A. (2023). Perspective vaccines for emerging viral diseases in farm animals. Clinical and Experimental Vaccine Research.

[bb0040] Almeida C.F., Faria M., Carvalho J., Pinho E. (2024). Contribution of nanotechnology to greater efficiency in animal nutrition and production. Journal of Animal Physiology and Animal Nutrition.

[bb0045] Al-Nemrawi N.K., Darweesh R.S., Al-Shriem L.A., Al-Qawasmi F.S., Emran S.O., Khafajah A.S., Abu-Dalo M.A. (2022). Polymeric nanoparticles for inhaled vaccines. Polymers.

[bb0050] Al-Sultan S.I., Hereba A.R.T., Hassanein K.M., Abd-Allah S.M., Mahmoud U.T., Abdel-Raheem S.M. (2022). The impact of dietary inclusion of silver nanoparticles on growth performance, intestinal morphology, caecal microflora, carcass traits and blood parameters of broiler chickens. Italian Journal of Animal Science.

[bb0055] Amor I.B., Hemmami H., Laouini S.E., Ahmed S., Mohammed H.A., Abdullah J.A.A., Alharthi F. (2024). Enhancing oxidant and dye scavenging through MgO-based chitosan nanoparticles for potential antioxidant coatings and efficient photocatalysts. Biomass Conversion and Biorefinery.

[bb0060] Angelopoulou M., Petrou P.S., Raptis I., Misiakos K., Livaniou E., Makarona E., Kakabakos S. (2021). Rapid detection of mozzarella and feta cheese adulteration with cow milk through a silicon photonic immunosensor. Analyst.

[bb0065] Anwar M., Muhammad F., Akhtar B. (2021). Biodegradable nanoparticles as drug delivery devices. Journal of Drug Delivery Science and Technology.

[bb0070] Arvidsson Segerkvist K., Hansson H., Sonesson U., Gunnarsson S. (2020). Research on environmental, economic, and social sustainability in dairy farming: A systematic mapping of current literature. Sustainability.

[bb0075] Baig N., Kammakakam I., Falath W. (2021). Nanomaterials: A review of synthesis methods, properties, recent progress, and challenges. Materials Advances.

[bb0080] Baker S., Volova T., Prudnikova S.V., Satish S., Prasad N. (2017). Nanoagroparticles emerging trends and future prospect in modern agriculture system. Environmental Toxicology and Pharmacology.

[bb0085] Bhagat S., Singh S. (2022). Nanominerals in nutrition: Recent developments, present burning issues and future perspectives. Food Research International.

[bb0090] Bhakat C., Mohammad A., Mandal D.K., Mandal A., Rai S., Chatterjee A., Dutta T.K. (2020). Readily usable strategies to control mastitis for production augmentation in dairy cattle: A review. Veterinary World.

[bb0095] Bravo-Ureta B.E., Wall A., Neubauer F. (2021). Dairy farming from a production economics perspective: An overview of the literature. Handbook of Production Economics.

[bb0100] Brito L.F., Bédère N., Douhard F., Oliveira H.R., Arnal M., Peñagaricano F., Miglior F. (2021). Genetic selection of high-yielding dairy cattle toward sustainable farming systems in a rapidly changing world. Animal.

[bb0105] Brohi R.D., Wang L., Talpur H.S., Wu D., Khan F.A., Bhattarai D., Huo L.J. (2017). Toxicity of nanoparticles on the reproductive system in animal models: A review. Frontiers in Pharmacology.

[bb0110] Chen M., Zhou S., Zhu Y., Sun Y., Zeng G., Yang C.C., Zhang W. (2018). Toxicity of carbon nanomaterials to plants, animals and microbes: Recent progress from 2015-present. Chemosphere.

[bb0115] Cheng W.N., Han S.G. (2020). Bovine mastitis: Risk factors, therapeutic strategies, and alternative treatments—A review. Asian-Australasian Journal of Animal Sciences.

[bb0120] Chick S., Ataei Kachouei M., Knowlton K., Ali M.A. (2024). Functionalized graphene-based biosensors for early detection of subclinical ketosis in dairy cows. ACS Applied Materials & Interfaces.

[bb0125] Chung H., Li J., Kim Y., Van Os J.M., Brounts S.H., Choi C.Y. (2020). Using implantable biosensors and wearable scanners to monitor dairy cattle’s core body temperature in real-time. Computers and Electronics in Agriculture.

[bb0130] Couto C., Almeida A. (2022). Metallic nanoparticles in the food sector: A mini-review. Foods.

[bb0135] Dadi R., Azouani R., Traore M., Mielcarek C., Kanaev A. (2019). Antibacterial activity of ZnO and CuO nanoparticles against gram positive and gram negative strains. Materials Science and Engineering: C.

[bb0140] De Faria L.V., Lisboa T.P., da Silva Campos N., Alves G.F., Matos M.A.C., Matos R.C., Munoz R.A.A. (2021). Electrochemical methods for the determination of antibiotic residues in milk: A critical review. Analytica Chimica Acta.

[bb0145] De Silva C., Nawawi N.M., Abd Karim M.M., Abd Gani S., Masarudin M.J., Gunasekaran B., Ahmad S.A. (2021). The mechanistic action of biosynthesised silver nanoparticles and its application in aquaculture and livestock industries. Animals.

[bb0150] Díaz-García V., Contreras-Trigo B., Rodríguez C., Coelho P., Oyarzún P. (2022). A simple yet effective preanalytical strategy enabling the application of aptamer-conjugated gold nanoparticles for the colorimetric detection of antibiotic residues in raw milk. Sensors.

[bb0155] Djearamane S., Xiu L.J., Wong L.S., Rajamani R., Bharathi D., Kayarohanam S., Selvaraj S. (2022). Antifungal properties of zinc oxide nanoparticles on Candida albicans. Coatings.

[bb0160] Dumlu B. (2024). Importance of Nano-sized feed additives in animal nutrition. Journal of Agricultural Production.

[bb0165] Dutta V., Verma R., Gopalkrishnan C., Yuan M.H., Batoo K.M., Jayavel R., Ghotekar S. (2022). Bio-inspired synthesis of carbon-based nanomaterials and their potential environmental applications: A state-of-the-art review. Inorganics.

[bb0170] Džermeikaitė K., Bačėninaitė D., Antanaitis R. (2023). Innovations in cattle farming: Application of innovative technologies and sensors in the diagnosis of diseases. Animals.

[bb0175] El-Gohary F.A., Abdel-Hafez L.J.M., Zakaria A.I., Shata R.R., Tahoun A., El-Mleeh A., Elmahallawy E.K. (2020). Enhanced antibacterial activity of silver nanoparticles combined with hydrogen peroxide against multidrug-resistant pathogens isolated from dairy farms and beef slaughterhouses in Egypt. Infection and Drug Resistance.

[bb0180] El-Sayed A., Kamel M. (2021). Bovine mastitis prevention and control in the post-antibiotic era. Tropical Animal Health and Production.

[bb0185] Ema M., Gamo M., Honda K. (2017). A review of toxicity studies on graphene-based nanomaterials in laboratory animals. Regulatory Toxicology and Pharmacology.

[bb0190] Gao J., He S., Nag A., Wong J.W.C. (2021). A review of the use of carbon nanotubes and graphene-based sensors for the detection of aflatoxin M1 compounds in milk. Sensors.

[bb0200] Ghosh S., Sikdar D., Bandyopadhyay K. (2022). Nanofortification: An emerging technique for enhancement of the functionality of foods with ample future scopes. Nanotechnology in Functional Foods.

[bb0205] Gonçalves D.R., Leroy J.L., Van Hees S., Xhonneux I., Bols P.E., Kiekens F., Marei W.F. (2021). Cellular uptake of polymeric nanoparticles by bovine cumulus-oocyte complexes and their effect on in vitro developmental competence. European Journal of Pharmaceutics and Biopharmaceutics.

[bb0210] Gulati S., Kumar S., Goyal K., Arora A., Varma R.S. (2022). Improving the air quality with functionalized carbon nanotubes: Sensing and remediation applications in the real world. Chemosphere.

[bb0215] Gulzar M.W., Khan M.K., Gulzar R., Suleman M., Hussain J., Hassan A., Haider Z. (2024). Nanotechnology application in overcoming the reproductive disorders in livestock: Nanotechnology in livestock reproduction. Letters in Animal Biology.

[bb0220] Gutiérrez P., Godoy S.E., Torres S., Oyarzún P., Sanhueza I., Díaz-García V., Coelho P. (2020). Improved antibiotic detection in raw milk using machine learning tools over the absorption spectra of a problem-specific nanobiosensor. Sensors.

[bb0225] Hamed S., Emara M. (2023). Antibacterial and antivirulence activities of acetate, zinc oxide nanoparticles, and vitamin C against E. Coli O157: H7 and P. Aeruginosa. Current Microbiology.

[bb0230] Hashem N.M., El-Sherbiny H.R., Fathi M., Abdelnaby E.A. (2022). Nanodelivery system for ovsynch protocol improves ovarian response, ovarian blood flow Doppler velocities, and hormonal profile of goats. Animals.

[bb0235] Hashem N.M., Gonzalez-Bulnes A. (2021). Nanotechnology and reproductive management of farm animals: Challenges and advances. Animals.

[bb0240] Hassan A.A., Mansour M.K., El Ahl R.M.S., El Hamaky A.M., Oraby N.H. (2020). Carbon nanomaterials for Agri-food and environmental applications, Micro and Nano Technologies.

[bb0245] Heydari Gharehcheshmeh M., Arianfar A., Mahdian E., Naji-Tabasi S. (2021). Production and evaluation of sweet almond and sesame oil nanoemulsion and their effects on physico-chemical, rheological and microbial characteristics of enriched yogurt. Journal of Food Measurement and Characterization.

[bb0250] Isibor P.O. (2024). Environmental Nanotoxicology: Combatting the minute contaminants.

[bb0255] Jiang M., Song Y., Kanwar M.K., Ahammed G.J., Shao S., Zhou J. (2021). Phytonanotechnology applications in modern agriculture. Journal of Nanobiotechnology.

[bb0260] Jing M., Zhang H., Li M., Mao Z., Shi X. (2021). Silver nanoparticle-decorated TiO2 nanotube array for solid-phase microextraction and SERS detection of antibiotic residue in milk. Spectrochimica Acta Part A: Molecular and Biomolecular Spectroscopy.

[bb0265] Kalashgarani M.Y., Babapoor A. (2022). Application of nano-antibiotics in the diagnosis and treatment of infectious diseases. Advances in Applied NanoBio-Technologies.

[bb0270] Kalińska A., Jaworski S., Wierzbicki M., Kot M., Radzikowski D., Smulski S., Gołębiewski M. (2023). Silver and copper nanoparticles as the new biocidal agents used in pre-and post-milking disinfectants with the addition of cosmetic substrates in dairy cows. International Journal of Molecular Sciences.

[bb0275] Kalpana R., Devasena T., Sudha S. (2020). Optoelectronic method of test for melamine adulteration in milk using paranitroaniline modified silver nanoparticles. International Conference on Nanotechnology.

[bb0280] Karthick G.S., Sridhar M., Pankajavalli P.B. (2020). Internet of things in animal healthcare (IoTAH): review of recent advancements in architecture, sensing technologies and real-time monitoring. SN Computer Science.

[bb0285] Khan M.Z.H. (2022). Recent biosensors for detection of antibiotics in animal derived food. Critical Reviews in Analytical Chemistry.

[bb0290] Kianfar E. (2021). Protein nanoparticles in drug delivery: Animal protein, plant proteins and protein cages, albumin nanoparticles. Journal of Nanobiotechnology.

[bb0295] Kim D.Y., Kadam A., Shinde S., Saratale R.G., Patra J., Ghodake G. (2018). Recent developments in nanotechnology transforming the agricultural sector: A transition replete with opportunities. Journal of the Science of Food and Agriculture.

[bb0300] Kozina A.M., Semkiv L.P. (2020). IOP Conference Series: Earth and Environmental Science (1–5).

[bb0305] Kumar H., Kuča K., Bhatia S.K., Saini K., Kaushal A., Verma R., Kumar D. (2020). Applications of nanotechnology in sensor-based detection of foodborne pathogens. Sensors.

[bib561] Yusof H.M., Rahman N.A.A., Mohamad R., Zaidan U.H., Samsudin A.A. (2022). Optimization of biosynthesis zinc oxide nanoparticles: Desirability-function based response surface methodology, physicochemical characteristics, and its antioxidant properties. OpenNano.

[bb0310] Kumar, R., Chowdhury, A., Rose, M. K., Sindhu, S., Syed, S. M., & Ghosh, M. (2024). Nanotechnology in prophylaxis of viral livestock diseases, *Nanotechnology theranostics in livestock diseases and management* (pp. 317–343). Singapore: Springer Nature Singapore.

[bb0315] Kumar V., Guleria P., Mehta S.K. (2017). Nanosensors for food quality and safety assessment. Environmental Chemistry Letters.

[bb0320] Lee M., Seo S. (2021). Wearable wireless biosensor technology for monitoring cattle: A review. Animals.

[bb0325] Liu R., Luo C., Pang Z., Zhang J., Ruan S., Wu M., Wang L., Sun T., Li N., Han L., Shi J. (2023). Advances of nanoparticles as drug delivery systems for disease diagnosis and treatment. Chinese Chemical Letters.

[bb0330] Lodhi F.L., Saleem M.I., Aqib A.I., Rashid I., Qureshi Z.I., Anwar M.A., Javaid M.K. (2021). Bringing resistance modulation to epidemic methicillin resistant S. Aureus of dairy through antibiotics coupled metallic oxide nanoparticles. Microbial Pathogenesis.

[bb0335] Luo M.X., Hua S., Shang Q.Y. (2021). Application of nanotechnology in drug delivery systems for respiratory diseases. Molecular Medicine Reports.

[bb0340] Malakar A., Kanel S.R., Ray C., Snow D.D., Nadagouda M.N. (2021). Nanomaterials in the environment, human exposure pathway, and health effects: A review. Science of the Total Environment.

[bb0345] Malhotra N., Lee J.S., Liman R.A.D., Ruallo J.M.S., Villaflores O.B., Ger T.R., Hsiao C.D. (2020). Potential toxicity of iron oxide magnetic nanoparticles: A review. Molecules.

[bb0355] Mekonnen G. (2021). Review on application of nanotechnology in animal health and production. Journal of Nanomedicine & Nanotechnology.

[bb0360] Michalak I., Dziergowska K., Alagawany M., Farag M.R., El-Shall N.A., Tuli H.S., Dhama K. (2022). The effect of metal-containing nanoparticles on the health, performance and production of livestock animals and poultry. Veterinary Quarterly.

[bb0370] Muhammad A., Hajian R., Yusof N.A., Shams N., Abdullah J., Woi P.M., Garmestani H. (2018). A screen printed carbon electrode modified with carbon nanotubes and gold nanoparticles as a sensitive electrochemical sensor for determination of thiamphenicol residue in milk. RSC Advances.

[bb0375] Neculai-Valeanu A.S., Ariton A.M. (2022). Udder health monitoring for prevention of bovine mastitis and improvement of milk quality. Bioengineering.

[bb0385] Omar M.E., Hassanein E.M., Shehabeldin A.M., Szenci O., El-Shereif A.A. (2025). Evaluating the impact of minimized GnRH and PGF2α analogues-loaded chitosan nanoparticles on ovarian activity and fertility of heat-stressed dairy cows. Pharmaceutics.

[bb0390] Ozogul Y., Karsli G.T., Durmuş M., Yazgan H., Oztop H.M., McClements D.J., Ozogul F. (2022). Recent developments in industrial applications of nanoemulsions. Advances in Colloid and Interface Science.

[bb0395] Panghal A., Chhikara N., Anshid V., Sai Charan M.V., Surendran V., Malik A., Dhull S.B. (2019). Nanobiotechnology in bioformulations.

[bb0400] Park H.G., Yeo M.K. (2016). Nanomaterial regulatory policy for human health and environment. Molecular & Cellular Toxicology.

[bb0405] Pereira T.D.S., dos Santos D.M., Andre R.D.S., Correa D.S. (2024). Zein/MnO2 nanosheet composites integrated with a smartphone for colorimetric sensors for on-site detection of adulterants in milk. ACS Applied Nano Materials.

[bb0410] Pérez-Hernández H., Pérez-Moreno A., Sarabia-Castillo C.R., García-Mayagoitia S., Medina-Pérez G., López-Valdez F., Fernández-Luqueño F. (2021). Ecological drawbacks of nanomaterials produced on an industrial scale: Collateral effect on human and environmental health. Water, Air, & Soil Pollution.

[bb0415] Poudineh M., Maikawa C.L., Ma E.Y., Pan J., Mamerow D., Hang Y., Kim S. (2021). A fluorescence sandwich immunoassay for the real-time continuous detection of glucose and insulin in live animals. Nature Biomedical Engineering.

[bb0425] Qazi H.J., Ye A., Acevedo-Fani A., Singh H. (2022). Impact of recombined milk systems on gastrointestinal fate of curcumin nanoemulsion. Frontiers in Nutrition.

[bb0430] Raya S.D.H.A., Hassan M.I., Farroh K.Y., Hashim S.A., Salaheldin T.A. (2016). Zinc oxide nanoparticles fortified biscuits as a nutritional supplement for zinc deficient rats. Journal of Nano Research.

[bb0435] Rehman S., Deeba F., ul Haq E., Adil M., Tanveer H.R., Ahmad S., Tayyab M. (2024). Complementary and alternative medicine: Nanotechnology-II.

[bb0440] Salama H.H., El-Sayed H.S., Kholif A.M., Edris A.E. (2022). Essential oils nanoemulsion for the flavoring of functional stirred yogurt: Manufacturing, physicochemical, microbiological, and sensorial investigation. Journal of the Saudi Society of Agricultural Sciences.

[bb0445] Saleem H., Zaidi S.J. (2020). Developments in the application of nanomaterials for water treatment and their impact on the environment. Nanomaterials.

[bb0450] Saleem H., Zaidi S.J., Alnuaimi N.A. (2021). Recent advancements in the nanomaterial application in concrete and its ecological impact. Materials.

[bb0455] Sarkar A., Dutta A. (2020). Challenges and opportunities of dairy sector in India Vis-à-Vis world: A critical review. Exploratory Animal & Medical Research.

[bb0460] Shalileh F., Sabahi H., Dadmehr M., Hosseini M. (2023). Sensing approaches toward detection of urea adulteration in milk. Microchemical Journal.

[bb0465] Shoaib M., Nawal A., Zámečník R., Korsakienė R., Rehman A.U. (2022). Go green! Measuring the factors that influence sustainable performance. Journal of Cleaner Production.

[bb0470] Singh B., Bhat A., Dutta L., Pati K.R., Korpan Y., Dahiya I. (2023). Electrochemical biosensors for the detection of antibiotics in milk: Recent trends and future perspectives. Biosensors.

[bb0475] Singh M. (2023). Nanosensor platforms for detection of milk adulterants. Sensors and Actuators Reports.

[bb0480] Singh P., Singh S., Nara S. (2021). Nanomaterials and nanotechnology: Biomedical, environmental, and industrial applications.

[bb0485] Singh R., Kumar N., Mehra R., Gupta S., Kumar H. (2020). UGC Sponsored National Conference on *Food Safety, Nutritional Security and Sustainability*.

[bb0490] Smykov I.T. (2020). The ELSI handbook of nanotechnology: Risk, safety, ELSI and commercialization.

[bb0495] Srilekha G.K.P., Tiwari H., Mketo N., Lakkakula J. (2022). Advances in dairy microbial products.

[bb0500] Suminda G.G.D., Bhandari S., Won Y., Goutam U., Pulicherla K.K., Son Y.O., Ghosh M. (2022). High-throughput sequencing technologies in the detection of livestock pathogens, diagnosis, and zoonotic surveillance. Computational and Structural Biotechnology Journal.

[bb0505] Tanveer Z.I., Huang Q., Xu T., Chen Y., Liu X., Han Z., Wu Y. (2023). Reduced graphene oxide-zinc iron oxide nanomaterial as selective dispersive solid-phase extraction sorbent for extraction and enrichment of aflatoxins from milk combined with UHPLC-MS/MS analysis. Microchemical Journal.

[bb0510] Tavares T.D., Antunes J.C., Padrão J., Ribeiro A.I., Zille A., Amorim M.T.P., Felgueiras H.P. (2020). Activity of specialized biomolecules against gram-positive and gram-negative bacteria. Antibiotics.

[bb0515] Van der Merwe D., Pickrell J.A. (2018). Veterinary toxicology.

[bb0520] Varun S., Daniel S.K., Gorthi S.S. (2017). Rapid sensing of melamine in milk by interference green synthesis of silver nanoparticles. Materials Science and Engineering: C.

[bb0525] Vejan P., Khadiran T., Abdullah R., Ahmad N. (2021). Controlled release fertilizer: A review on developments, applications and potential in agriculture. Journal of Controlled Release.

[bb0530] Vekariya S.J., Rajput M.B., Vataliya P.H. (2021). Prospects of dairy farming in India: A review. International Journal of Current Microbiology and Applied Sciences.

[bb0535] Vuppu S., Chavda V.P., Mishra T., Punetha S., Sharma N., Kamaraj S., Raj V. (2024). Nanocarrier vaccines: Biopharmaceutics-based fast track development.

[bb0540] Vuran B., Ulusoy H.I., Sarp G., Yilmaz E., Morgül U., Kabir A., Soylak M. (2021). Determination of chloramphenicol and tetracycline residues in milk samples by means of nanofiber coated magnetic particles prior to high-performance liquid chromatography-diode array detection. Talanta.

[bb0545] Woldeamanuel K.M., Kurra F.A., Roba Y.T. (2021). A review on nanotechnology and its application in modern veterinary science. International Journal of Nanomaterials, Nanotechnology and Nanomedicine.

[bb0550] Yadav A., Yadav K., Ahmad R., Abd-Elsalam K.A. (2023). Emerging frontiers in nanotechnology for precision agriculture: Advancements, hurdles and prospects. Agrochemicals.

[bb0555] Yadav P., Yadav A.B., Gaur P., Mishra V., Huma Z.I., Sharma N., Son Y.O. (2022). Bioengineered ciprofloxacin-loaded chitosan nanoparticles for the treatment of bovine mastitis. Biomedicines.

[bb0560] Yao Y., Zhang Z., Yang Z. (2023). The combination of vaccines and adjuvants to prevent the occurrence of high incidence of infectious diseases in bovine. Frontiers in Veterinary Science.

